# Validating hidden Markov models for seabird behavioural inference

**DOI:** 10.1002/ece3.11116

**Published:** 2024-03-04

**Authors:** Rebecca A. Akeresola, Adam Butler, Esther L. Jones, Ruth King, Víctor Elvira, Julie Black, Gail Robertson

**Affiliations:** ^1^ School of Mathematics and Maxwell Institute for Mathematical Sciences University of Edinburgh Edinburgh UK; ^2^ Biomathematics & Statistics Scotland Edinburgh UK; ^3^ Joint Nature Conservation Committee Aberdeen UK

**Keywords:** conservation, GPS data, movement data, movement modelling, visual tracking

## Abstract

Understanding animal movement and behaviour can aid spatial planning and inform conservation management. However, it is difficult to directly observe behaviours in remote and hostile terrain such as the marine environment. Different underlying states can be identified from telemetry data using hidden Markov models (HMMs). The inferred states are subsequently associated with different behaviours, using ecological knowledge of the species. However, the inferred behaviours are not typically validated due to difficulty obtaining ‘ground truth’ behavioural information. We investigate the accuracy of inferred behaviours by considering a unique data set provided by Joint Nature Conservation Committee. The data consist of simultaneous proxy movement tracks of the boat (defined as visual tracks as birds are followed by eye) and seabird behaviour obtained by observers on the boat. We demonstrate that visual tracking data is suitable for our study. Accuracy of HMMs ranging from 71% to 87% during chick‐rearing and 54% to 70% during incubation was generally insensitive to model choice, even when AIC values varied substantially across different models. Finally, we show that for foraging, a state of primary interest for conservation purposes, identified missed foraging bouts lasted for only a few seconds. We conclude that HMMs fitted to tracking data have the potential to accurately identify important conservation‐relevant behaviours, demonstrated by a comparison in which visual tracking data provide a ‘gold standard’ of manually classified behaviours to validate against. Confidence in using HMMs for behavioural inference should increase as a result of these findings, but future work is needed to assess the generalisability of the results, and we recommend that, wherever feasible, validation data be collected alongside GPS tracking data to validate model performance. This work has important implications for animal conservation, where the size and location of protected areas are often informed by behaviours identified using HMMs fitted to movement data.

## Graphical abstract

Behaviours inferred from telemetry data using hidden Markov models (HMMs) are not typically validated due to difficulty obtaining ‘ground truth’ behavioural information. Our study is unique in that we investigate the accuracy of HMM‐inferred behaviours by considering movement (visual tracks) and observed behavioural data sets recorded at the same time frequency provided by Joint Nature Conservation Committee. We show that HMMs fitted to tracking data can accurately identify important conservation‐relevant behaviours.

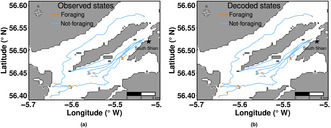

## 1 INTRODUCTION

Seabirds are key indicators of marine environmental health (Lascelles et al., 2012; Parsons et al., 2008) but are the most threatened and anthropogenically pressured group of birds globally (Croxall et al., 2012). Threats, including invasive species at breeding colonies, climate change, over‐fishing and offshore renewable developments, have resulted in a global decline in seabird populations of 70% over the last five decades (Vulcano et al., 2021). In the United Kingdom, some species of seabirds (e.g. Northern fulmar [*Fulmarus glacialis*], little tern [*Sternula albifrons*], European shag [*Phalacrocorax aristotelis*], Arctic skua [*Stercorarius parasiticus*] and black‐legged kittiwake [*Rissa tridactyla*]) have continued to decline (JNCC, 2021). Of the 25 seabird species that regularly breed in the United Kingdom, 24 are listed as Red or Amber on the UK's Birds of Conservation Concern (Stanbury et al., 2021). Under the Habitats Directive (EC/92/43) and Birds Directive (EC/79/409), Special Protection Areas (SPA) are established to form the Natura 2000 network, which protects species and habitats (European‐Commission et al., 2008). Although SPAs have historically been restricted to small areas focused on seabird breeding colonies, recent extensions and new classifications in the marine environment have expanded the SPA network across the United Kingdom (JNCC, 2020). Seabirds are restricted to central place foraging during the breeding season. Therefore, understanding at‐sea behaviour, including characterising important foraging areas, is vital to ensure adequate protection measures are in place to prevent further population decline.

Seabird tracking studies, where individuals are tagged using biologging technology, are an effective way to understand space use and behaviour (Bennison et al., 2018; Lascelles et al., 2012; Wakefield et al., 2017). Technological advances have accelerated the availability of biologging information from devices such as Global Positioning System (GPS) transmitters, accelerometers, conductivity‐temperature‐depth (CTD) tags and harmonic radar trackers (Cooke et al., 2004). Telemetry data provides information on animal locations at discrete intervals but does not provide direct information about the underlying behaviour of the tagged animals. To infer behavioural states such as foraging, flying and resting from movement data, hidden Markov Models (HMMs) have been widely used (Langrock et al., 2012; McClintock, 2021; McKellar et al., 2015; Morales et al., 2004; Patterson et al., 2009). HMMs are time series models with observation and state processes where the latent (unobserved) states are often interpreted as activities or behaviours relating to the ecology of the species (Langrock et al., 2012). Ecologically inferred behaviours post‐analysis, simplified to *inferred behaviours* hereafter, can be used to inform conservation decision‐making, for example, the size and location of protected areas.

One limitation of using inferred behaviours to inform conservation‐relevant decision‐making is the difficulty in validating models using ground truth data. Some studies have attempted to validate inferred behaviours from movement data, such as Joo et al. (2013), which validated the behaviour of fishing vessels using ground truth data recorded by onboard observers. Bennison et al. (2018) and Conners et al. (2021) also validated inferred behaviours of northern gannet (*Morus bassanus*) and albatross using behaviours from depth recorder and sensors as ground truth data respectively. However, depth recorders and sensors are also proxies for ground truth data with their own error structures. Overall, little research has focused on evaluating the performance of HMMs fitted to animal movement data through data validation because contemporaneous behavioural observations on tracked individuals can be challenging to collect, particularly in featureless environments, such as open ocean (Joo et al., 2013). To examine the performance of HMMs fitted to movement data, we consider a unique data set provided by the Joint Nature Conservation Committee (JNCC) and obtained via the visual tracking of terns (*Sterna* spp.) using a rigid‐hulled inflatable boat. A visual tracking method developed by Perrow et al. (2011) was conducted at several tern breeding colonies across the United Kingdom during chick‐rearing and incubation in different years (Wilson et al., 2014). Proxy movement data, corresponding to the GPS location of the boat, and the observed behavioural data of the terns directly recorded by the observers on the boat were collected at the same time frequency.

First‐hand behavioural data of seabirds such as that collected by Wilson et al. (2014) is generally not feasible to collect directly alongside GPS tracking location data. We consider terns as a case study to examine the performance of HMMs for behavioural inference. To the best of our knowledge, this is the first study to validate inferred behaviours from movement data using observed behavioural data of seabirds. Our study aims to leverage the rare opportunity provided by the unique JNCC data set to (i) examine whether boat locational data are an adequate proxy of tern movement data in relation to inferred behaviours and (ii) validate inferred behaviours of seabirds from HMMs fitted to boat locational data using behavioural observations of seabirds taken by the observes from the boat.

## 2 MATERIALS AND METHODS

### 2.1 Study species and sites

**FIGURE 1:**
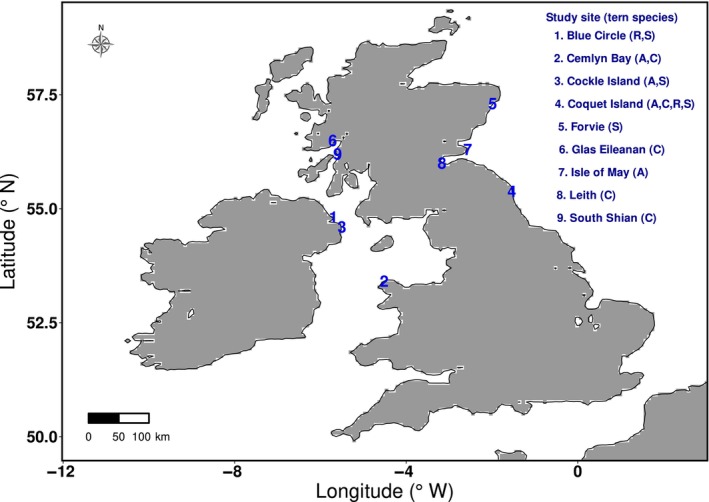
Study sites consisting of nine tern breeding colonies in the United Kingdom. A, Arctic; C, Common; R, Roseate; S, Sandwich tern.

This study investigates the movement behaviour of four tern (*Sterna* spp.) species: Arctic (*Sterna paradisaea*), common (*S. hirundo*), Sandwich (*S. sandvicensis*) and roseate (*S. dougallii*). Arctic terns tend to breed in coastal areas in the north and west of the United Kingdom, with 80% occurring in Shetland, Orkney and the Outer Hebrides. Common terns have a widespread coastal distribution around the United Kingdom and also nest in small colonies inland along rivers and islets. Sandwich terns congregate in several large colonies, and most roseate terns breed on Rockabill, Ireland, with some pairs occasionally breeding in south‐east Scotland, Norfolk and Hampshire (Wilson et al., 2014). Study sites comprised of nine breeding colonies across the United Kingdom (Figure 1): Blue Circle ($54^{{}^{\circ}}49^{\prime }\;N$, $5^{{}^{\circ}}46^{\prime }\;W$) and Cockle Island ($54^{{}^{\circ}}40^{\prime }\;N$, $5^{{}^{\circ}}37^{\prime }\;W$) in Northern Ireland; Cemlyn Bay ($53^{{}^{\circ}}24^{\prime }\;N$, $4^{{}^{\circ}}30^{\prime }\;W$) in North Wales; Glas‐Eileanan Island ($56^{{}^{\circ}}49^{\prime }\;N$, $5^{{}^{\circ}}71^{\prime }\;W$), Forvie ($57^{{}^{\circ}}18^{\prime }\;N$, $1^{{}^{\circ}}58^{\prime }\;W$), Isle of May ($56^{{}^{\circ}}10^{\prime }\;N$, $2^{{}^{\circ}}32^{\prime }\;W$), Leith ($55^{{}^{\circ}}96^{\prime }\;N$, $3^{{}^{\circ}}16^{\prime }\;W$) and South Shian ($56^{{}^{\circ}}46^{\prime }\;N$, $5^{{}^{\circ}}36^{\prime }\;W$) in Scotland; and Coquet Island ($55^{{}^{\circ}}20^{\prime }\;N$, $1^{{}^{\circ}}32^{\prime }\;W$) in England.

Terns are ground‐nesting colonial breeders, raising one brood each breeding season (May–June) and laying a clutch of one to three eggs. While breeding adult terns are central place foragers throughout the breeding season, they are particularly restricted during chick‐rearing when they must return regularly to provision their chicks, and adults spend up to 80% of their time foraging (Thaxter et al., 2012). Sandwich terns are specialist predators that can exploit clupeids and sandeels from deeper water, potentially due to their wider foraging range. Likewise, roseate terns are specialists who also forage by plunge diving to depth, catching prey items of predominately sandeels, herring and sprat. Common terns are generalist predators and prey items include invertebrates, clupeids, sandeels and gadoids. Arctic terns forage using several techniques but are heavily dependent on sandeel and changes in prey availability can affect their breeding success (Eglington & Perrow, 2014).

### 2.2 Visual tracking data

Visual tracking data were collected using a technique developed by Perrow et al. (2011) and detailed in Wilson et al. (2014). We summarise the protocol as follows: The visual tracking of terns was conducted during chick‐rearing (June and July) and incubation (early May to mid‐June) between 2009 and 2011. Rigid hull inflatable boats used for the visual tracking were operated by different skippers across the study sites. The boats were kept c. 50–200 m from terns while an individual was tracked to avoid disturbing the birds and affecting their behaviour. Longitude and latitude of the boats were recorded using an onboard GPS device set to a 1 s sampling frequency. Individuals were tracked on return foraging trips from their breeding colony. One observer maintained constant sight of the tracked individual, while another recorded behavioural information.

An ethogram of continuous flight behaviours and instantaneous foraging events was provided to each observer, and the timing of each behaviour was recorded (Wilson et al., 2014). Flight behaviours were categorised as active search, transit search and direct flight. Direct flight was defined as a clear and consistent direction with fast flight usually returning to the colony with food. An active search was defined as an erratic flight course actively searching for food, which may include instances of diving and surface feeding. It is hypothesised that for a direct flight, terns have a fixed location in view and fly in a clear and consistent direction, whereas for transit search, they may change direction but not erratically to search for food (Wilson et al., 2014). As a result, direct flight and transit search were defined as observed not‐foraging behaviour while an active search was defined as observed foraging behaviour. These behavioural data are used as the validation data in the study.

**FIGURE 2:**
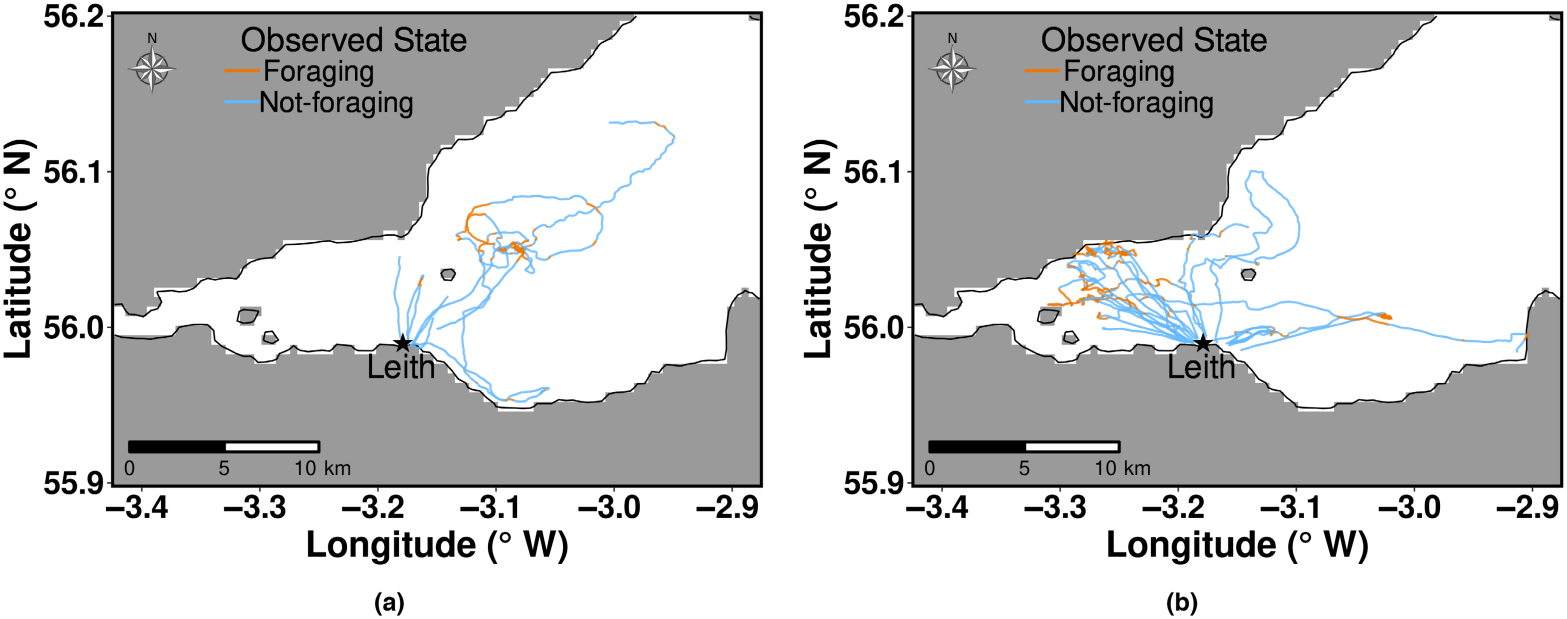
Visual tracks of common terns coloured with observed behavioural states during (a) incubation and (b) chick‐rearing period from Leith dock, 2010.

The location of each observed behaviour was calculated from the boat's GPS track log. Unique IDs were assigned to the data of individual terns tracked in each colony. In 2009 and 2011, tracking only took place during chick‐rearing. In 2010, tracking was conducted during chick‐rearing and incubation periods. Figure 2 provides an example of visual tracks for the two breeding seasons. The data combined both complete and incomplete tracks of terns. The track of terns was considered complete if individual terns were tracked leaving and returning to the colony. Incomplete tracks were terns that could not be successfully followed back to the colony.

Reasons for incomplete tracks could be individuals flying faster than the boat could follow, flying over a physical obstruction that prevented the boat from following, observers confusing the tracked individuals with other terns, or insufficient fuel in the boat (Perrow et al., 2011).

Visual tracks for which (i) a single observed behaviour was recorded throughout the tracking trip and (ii) total tracking time that did not exceed 1 min are omitted (see Table S1 for a summary of visual tracks used, Supporting information). GPS coordinates of the boat are subsequently converted into step length (km) and turning angle (radians). These calculated metrics potentially provide information about tern behaviour. For example, foraging behavioural activities are typically characterised by slow and tortuous flight, indicating smaller step lengths and low directional persistence in turnings. In contrast, not‐foraging behavioural activities are generally characterised by longer step lengths and high directional persistence in turnings (Morales et al., 2004).

### 2.3 Visual tracks as a proxy for tern tracks

Given that the boat followed at a distance c. 50–200 m from the tracked terns, we investigate how well boat tracks replicate the movement of tracked individuals using additional information on the animal's recorded position in relation to the boat. For a subset of tracks recorded at the Coquet Island colony during the chick‐rearing in 2009, additional data were also collected corresponding to the distance and bearing of the tern from the boat, thus permitting the reconstruction of the (approximate) longitude and latitude location of the tern.

Mathematically, let ${\mathrm{Lon}}_{\text{boat}}$ and ${\mathrm{Lat}}_{\text{boat}}$ denote the boat's longitude and latitude position and the bearing and distance of the boat to the tern be indicated by ‘bearing’ and ‘distance’ respectively. Then the corresponding tern longitude and latitude (${\mathrm{Lon}}_{\text{tern}}$, ${\mathrm{Lat}}_{\text{tern}}$) are given by:

$$\begin{array}{l} {\mathrm{Lat}}_{\text{tern}}\approx \arcsin\left(\sin\left({\mathrm{Lat}}_{\text{boat}}\right)\times \cos\left(\text{distance}/R\right)+\cos\left({\mathrm{Lat}}_{\text{boat}}\right)\times \sin\left(\text{distance}/R\right)\times \cos\left(\text{bearing}\right)\right),\\ {\mathrm{Lon}}_{\text{tern}}\approx {\mathrm{Lon}}_{\text{boat}}+\text{atan}2\left(y,x\right), \end{array}$$

where

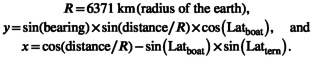




We compare (i) boat tracks and approximate tern tracks and (ii) the distribution of step length and angles corresponding to the boats and approximate tern tracks to determine whether the former can be used as an approximation for the movement of individual terns. We then model the boat and approximated tern tracking data using HMMs to account for the different movement patterns dependent on the (unknown) underlying behavioural states. We then extract the inferred behavioural states from models fitted to both data sets and create a confusion matrix to assess differences and similarities in inferred states.

### 2.4 Hidden Markov model (HMM)

**FIGURE 3:**
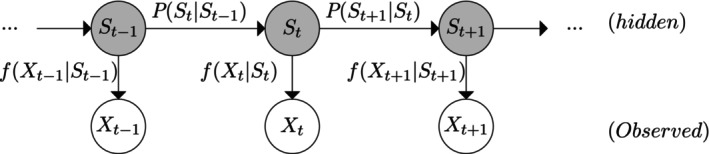
Graphical representation of a HMM where $S_{t}$ and $X_{t}$ denotes the state and observed process.

A HMM (Figure 3) is a time series model with an observed component, $X_{t}$, driven by an underlying latent component known as the state process, $S_{t}$. The latter, $S_{t}$, takes a value on a finite set of *N* possible values and is assumed to be a first‐order Markov chain with the state transition probability $\gamma_{\mathrm{ij}}=P\left(S_{t}=j|S_{t-1}=i\right)$. The observed component, $X_{t}$, which can be univariate or multivariate are conditionally independent given the underlying states (and parameters), and assumed to be regularly spaced in time, *t*, with the associated observation process distribution $f\left(X_{t}|S_{1},\ldots ,S_{T}\right)=f\left(X_{t}|S_{t}\right)$ at any given time *t*.

HMMs are suitable for fitting to the visual tracking data since observations are collected at a regularly spaced interval, and for each time *t*, we specify N=2 discrete states corresponding to foraging $\left(S_{t}=1\right)$ and not‐foraging $\left(S_{t}=2\right)$.

Each observed data point, $X_{t}$, is bi‐dimensional, consisting of the step length (km), $r_{t}$ and the turning angle (radians), $\psi_{t}$. At each time *t*, the distribution of $X_{t}$ is conditional on the current hidden state, $S_{t}$, such that

(1) $$f_{j}\left(X_{t}\right)=\left(f\left(r_{t}|s_{t}=j\right),f\left(\psi_{t}|s_{t}=j\right)\right)\;\text{for}\;j=1,\ldots ,N,$$

where $r_{t}$ is modelled from a gamma distribution with parameters mean, μ, and standard deviation, σ, that is, $r_{t}|\left(S_{t}=j\right)∼\text{Gamma}\left(\mu_{j},\sigma_{j}\right)$, and $\psi_{t}$ is modelled from a von Mises distribution with parameters mean, ρ, and concentration, κ, that is, $\psi_{t}|\left(S_{t}=j\right)∼\mathrm{von}‐\text{Mises}\left(\rho_{j},\kappa_{j}\right)$. We assume the distributions are independent for each time *t*, conditional on the underlying state $S_{t}$.

The corresponding likelihood of a HMM is a function of the following parameters: (i) δ: $\left(1\times N\right)$ vector of initial state distribution given as $\delta =\left(P\left(S_{1}=1\right),\ldots ,P\left(S_{1}=N\right)\right)$ (ii) Γ: $\left(N\times N\right)$ matrix of transition probabilities given as

(2) $$\Gamma =\left(\begin{array}{ccc} \gamma_{11} & \ldots & \gamma_{1N}\\ \vdots & \ddots & \vdots \\ \gamma_{N1} & \ldots & \gamma_{\mathrm{NN}} \end{array}\right),$$

(iii) $P\left(X_{t}\right)$: $\left(N\times N\right)$ diagonal matrix corresponding to the observation process given as

(3) $$P\left(X_{t}=x_{t}\right)=\left(\begin{array}{ccc} f\left(X_{t}=x_{t}|S_{t}=1\right) & \ldots & 0\\ \vdots & \ddots & \vdots \\ 0 & \ldots & f\left(X_{t}=x_{t}|S_{t}=N\right) \end{array}\right).$$

The general likelihood function of a HMM is then given by

(4) $$ℒ=P\left(X_{t}=x_{t}|\theta \right)=\delta \Gamma P\left(x_{1}\right)\ldots \Gamma P\left(x_{T}\right)1,$$

where **1** is a column vector of length *N* with all entries equal to 1. We estimate these parameters, $\theta =\left\{\delta ,\gamma ,\mu ,\sigma ,\rho ,\kappa \right\}$, via maximum likelihood estimation and obtain the most likely state sequence using the Viterbi algorithm (Zucchini et al., 2016). We used the R package momentuHMM (McClintock & Michelot, 2018) for fitting HMMs to the boat tracks since the package is widely used by scientists studying animal movement. We followed the guidance outlined in Michelot et al. (2016) to specify initial starting values for the model parameters.

### 2.5 HMMs specification and selection

Boat GPS locations were recorded at 1 s intervals and do not have missing data. The completeness of the data means it is possible to use the recorded positions directly without the need to standardise the recording frequency by interpolating in time and space. Seabirds have been shown to vary their behaviour and area use at different breeding stages, travelling further from the colony to rich foraging grounds during incubation and remaining closer to the colony to feed chicks during chick‐rearing (Robertson et al., 2014). As behaviour is expected to differ between the two periods, we expect model parameters to differ. We consider different models by varying model parameters for each tern species at each colony during the two periods, summarised in Table 1.

**TABLE 1:** HMMs (Models 0–6) fitted to the boat tracking data across study sites during incubation and chick‐rearing. Covariate = Euclidean distance of the boat to the study site.

Models	Pooling effect		Covariate effect	
	State process	Observed process	State process	Observed process
0	✓	✓	✗	✗
1	✗	✓	✗	✗
2	✓	✗	✗	✗
3	✗	✗	✗	✗
4	✓	✓	✓	✗
5	✓	✓	✗	✓
6	✓	✓	✓	✓

Model 0, the base model, specifies that the state and observed processes are completely pooled across the individual visual tracks so that the model parameters are assumed to be the same for all individuals. Model 1 assumes a unique transition probability matrix parameter, Γ, for each individual by removing the pooling effect on the state process. Model 2 assumes unique step length parameters for each individual track by removing the pooling effect on the observed process across tracks. The pooling effect on both state and observed process is not included in Model 3.

The Euclidean distance of the boat to the colony was included as an environmental covariate on the state process in Model 4, the observed process in Model 5 and both processes in Model 6. The parameters associated with the observed and state process are pooled across individual tracks for Models 4, 5 and 6 respectively. For seabird species in the breeding season, distance to colony will typically be a dominant factor in determining movement and spatial distributions at‐sea (e.g. Wakefield et al., 2017), because adults need to return to the colony regularly in order to feed their chicks. It is therefore natural to include the Euclidean distance of the boat to the colony as a potential covariate to describe both the probability of transition between states and the distribution of step length and turning angle within each state and the energetic cost of travelling to a particular location from the breeding colony (Wilson et al., 2014). We assume a simple logistic regression model to include the covariate for the state process in a 2‐state HMM (Models 4 and 6). Let *c* denote the covariate for the 2‐state HMM, we set

(5) $$\gamma_{\mathrm{ij}}=\frac{\exp\left(\eta_{\mathrm{ij}}\right)}{1+\exp\left(\eta_{\mathrm{ij}}\right)}\;\text{for}\;i,j=1,2,$$

where

(6) $$\left\{\begin{array}{l} \eta_{12}=\beta_{0}^{\left(12\right)}+\beta_{1}^{\left(12\right)}c_{12},\\ \eta_{21}=\beta_{0}^{\left(21\right)}+\beta_{1}^{\left(21\right)}c_{21},\\ \eta_{11}=\eta_{22}=0, \end{array}\right.$$

and $\beta_{0}$, $\beta_{1}$ corresponds to the intercept and the regression parameter of the covariate respectively. For the observed process (in Models 5 and 6), the covariate is included in the mean of the step length as $\mu =\exp\left(\beta_{0}+\beta_{1}c_{\mu }\right)$ and included in the standard deviation of the step length as $\sigma =\exp\left(\beta_{0}+\beta_{1}c_{\sigma }\right)$. Model selection was performed using the Akaike information criterion (AIC) (Burnham & Anderson, 2002).

### 2.6 Model validation

The validation data consist of the observed behaviours of visually tracked terns. The inferred behavioural states from HMMs and validation data are assumed to be binary classifications: foraging and not‐foraging. Common evaluation metrics for binary classification tasks include confusion matrix accuracy, F1‐score, area under a ROC curve and logarithmic loss (Hossin & Sulaiman, 2015). We observed an imbalance in the observed behavioural state distribution. In particular, we identified an unbalanced classification for some breeding colonies such as Cemlyn, Isle of May and Leith. Furthermore, we note that the positive class‐foraging behavioural state is less prevalent in the study but is of an increased interest as it is this state that helps to identify tern foraging areas. We use the F1‐score metric to validate behavioural states of visually tracked terns inferred from HMMs as it aims to address the problem of imbalanced classes. In particular, the F1‐score metric corrects for the imbalance using the harmonic mean between the precision and recall. The (i) positive predictive value (PPV or precision), which is the proportion of correct positives identified from all the predicted positives is calculated as

(7) $$\mathrm{PPV}=\frac{\text{number of true positive}}{\text{number of true positive}+\text{number of false positive}}$$

and (ii) true positive rate (TPR or recall), which is the proportion of the positives that are predicted correctly is expressed as

(8) $$\mathrm{TPR}=\frac{\text{number}\;\text{of}\;\text{true}\;\text{positive}}{\text{number}\;\text{of}\;\text{true}\;\text{positive}+\text{number}\;\text{of}\;\text{false}\;\text{negative}}$$

Using Equations (7) and (8), the F1‐score is calculated as

(9) $$F1‐\text{score}=2\left(\frac{{\mathrm{PPV}}^{*}\mathrm{TPR}}{\mathrm{PPV}+\mathrm{TPR}}\right)$$

We also report the negative predictive value (NPV), which is the percentage of correct not‐foraging behavioural states of all the decoded not‐foraging states expressed as

(10) $$\mathrm{NPV}=\frac{\text{number}\;\text{of}\;\text{true}\;\text{negative}}{\text{number}\;\text{of}\;\text{true}\;\text{negative}+\text{number}\;\text{of}\;\text{false}\;\text{negative}}$$

Although the F1‐score is a good validation metric, it does not account for how close the decoded behavioural state is to the observed behavioural state. However, the logarithmic loss metric, which is based on probability, does account for the uncertainty in the predicted classification (Hossin & Sulaiman, 2015). Thus, we also consider the logarithmic loss for the fitted HMMs to account for the uncertainty of the decoded behavioural state. We use the observed behavioural states at each point, $y_{i}$, and the predicted probabilities of locally decoded behavioural state, $q_{i}$, to calculate the logarithmic loss metric as

(11) $$\mathrm{Log}\;\text{loss}\left(y,q\right)=-\frac{1}{n}\sum_{i=1}^{n}\left[y_{i}\log\left(q_{i}\right)+\left(1-y_{i}\right)\log\left(1-q_{i}\right)\right]$$

where *n* is the number of observations. The fitted model with the lowest log‐loss value is deemed optimal for this criteria, and we report the F1‐score, PPV and TPR corresponding to the optimal HMM for each species and colony site.

In addition to the validation metrics, we (i) compare the observed behavioural data of each tern species to their respective decoded behavioural data obtained from optimal HMMs across breeding colonies, (ii) identify all foraging events from the observed behavioural data (where a foraging event is a bout within which only observed foraging behavioural states are recorded), (iii) note the decoded foraging behaviour from optimal HMMs that falls within the window of each observed foraging event as correctly inferred and (iv) calculate the proportion of observed foraging events where optimal HMMs identify (a) less than 25% (0% exclusive), (b) 25%–49%, (c) 50%–74% and (d) at least 75% of the observed foraging behavioural states. We also obtain the proportion of observed foraging events completely missed from the foraging behavioural states inferred from optimal HMMs (i.e. observed foraging events where the model infers foraging at 0% of the time points).

## 3 RESULTS

### 3.1 Assessment of visual tracking data as a proxy for tern movement data

**FIGURE 4:**
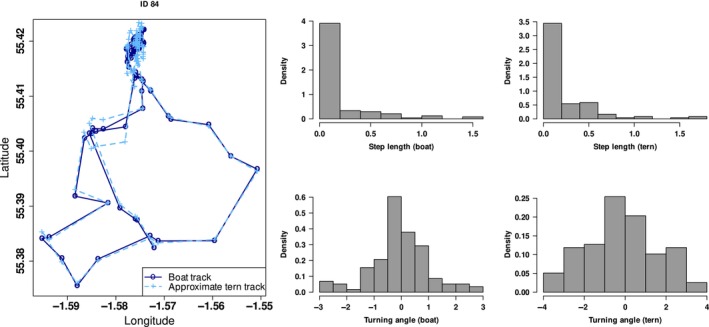
Approximate roseate tern tracks and boat tracks from Coquet Island, 2009 (column 1). Histogram showing the distribution of step length (km) and turning angles (radians) from boat tracks (column 2) and from approximate roseate tern tracks (column 3).

Reported results are based on visual tracking conducted at the Coquet Island colony during the chick‐rearing period in 2009. We compare the boat locations to the associated inferred movement track of nine terns and distributions of the derived step lengths and turning angles. Typical foraging movement patterns generated by the boat and inferred tern tracks are provided in Figure 4 (and Figures S1–S4). There are strong similarities between the locations (as would be expected given the boats were following the birds) and step length distributions. However, there appear to be more substantial differences with the turning angle distributions (lower panel of columns 2 and 3 in the figures). The latter difference can be explained by the bird making quicker turns compared to the boat, which has smoother turning movements.

**FIGURE 5:**
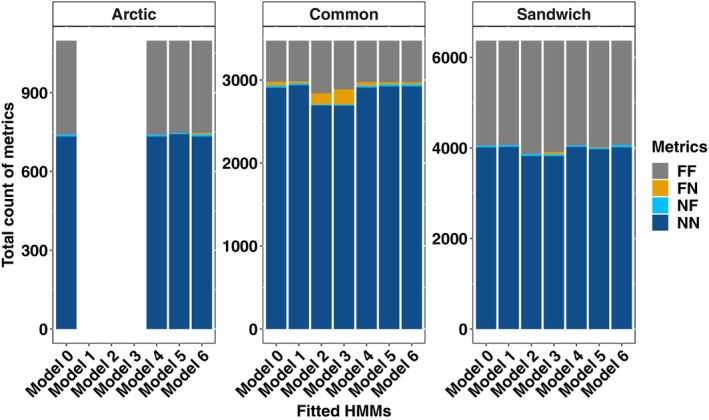
Confusion matrix metrics of behavioural states inferred from HMMs fitted to the boat and approximate location data of one Arctic, two common and five Sandwich terns from Coquet colony during chick‐rearing, 2009. FF = true positive (foraging in boat and tern location), FN = false negative (foraging in boat and not‐foraging in tern location), NF = false positive (not‐foraging in boat and foraging in tern location) and NN = true negative (not‐foraging in boat and tern location). Models 1, 2 and 3 require a minimum of two terns; hence, there is no bar for Arctic tern.

We fitted HMMs to both boat and inferred tern location data. Comparison of HMMs using locations of the boat and inferred locations of the tern are very similar. We observed little difference in the inferred behavioural states when using boat location to approximate the location of the tern. The confusion matrix metrics in Figure 5 indicate that the proportions of true positives and true negatives when comparing behaviours derived from fitting HMMs to boat and inferred tern locations against each other are higher than those of false negatives and false positives. This implies that majority of the time, foraging behavioural states decoded by HMMs from the boat locations correspond to foraging behavioural states decoded by HMMs from approximate tern locations with very few instances of cases where decoded behavioural states differ from both location data. Thus, the boat locations are adequate for HMMs in this case.

### 3.2 Validating HMM‐inferred behavioural states

Reported results are based on HMMs deemed optimal (i.e. HMMs with the lowest log‐loss value). Tables 2 and 3 present the summarised results of 2‐state HMMs fitted to the visual tracking data during chick‐rearing and incubation (see Tables S2–S4 for additional results).

**TABLE 2:** Validation results of 2‐state HMMs fitted to visual tracking data of terns during chick‐rearing.

Chick‐rearing		HMM deemed optimal (i.e. based on lowest log‐loss value)		Validation metrics (%)		
Colony	Species	Model	Model description	PPV	TPR	F1‐score
Coquet	Common	0	Complete pool	88.44	79.19	83.56
Glas Eileanan				84.21	73.49	78.48
Coquet	Arctic	1	No pool on Γ	66.84	61.75	64.19
Isle of May				86.77	70.65	77.88
Blue Circle	Sandwich	2	No pool on step	80.84	78.36	79.58
Leith	Common	2	No pool on step	74.05	70.19	72.07
Cockle	Sandwich	3	No pool on Γ and step	84.85	91.11	87.87
Coquet				86.90	74.77	80.38
Forvie				65.15	79.90	71.77
Cemlyn	Arctic	4	Covariate on Γ	98.91	58.92	73.85
Coquet	Roseate			68.66	86.06	76.38
South Shian	Common			70.51	90.64	79.32
Cemlyn	Common	6	Covariate on Γ and step	71.93	81.21	76.29

*Note*: Γ = transition probability matrix, covariate = Euclidean distance of boat to colony.

**TABLE 3:** Validation results of 2‐state HMMs fitted to visual tracking data of terns during incubation.

Incubation		HMM deemed optimal (i.e. based on lowest log‐loss value)		Validation metrics (%)		
Colony	Species	Model	Model Description	PPV	TPR	F1‐score
Leith	Common	0	Complete pool	61.00	60.29	60.64
Blue Circle	Roseate	4	Covariate on Γ	82.90	60.62	70.03
Cockle	Arctic	5	Covariate on step	60.11	68.63	64.08
Cockle	Sandwich	6	Covariate on Γ and step	59.54	49.65	54.15
Isle of May	Arctic			63.88	32.20	42.82

*Note*: Γ = transition probability matrix, covariate = Euclidean distance of boat to colony.

Correctly decoded foraging states relative to total decoded foraging states ranged from 65% to 98% during chick‐rearing. We note that correct decoded foraging states relative to total observed foraging states ranged from 70% to 91% except for Arctic terns from Cemlyn and Coquet study sites with 58% and 61% respectively. Overall, the performance of HMMs as measured by the F1‐score in correctly inferring behavioural states during chick‐rearing is at least 71% across study sites except for Arctic terns in Coquet, with a percentage of 64%. Validation of HMM results for incubation data shows a low performance compared to models fitted to chick‐rearing data in inferring behavioural states. For example, we recorded at least 70% for only one roseate tern visually tracked at the Blue Circle colony during incubation. The overall low performance during this breeding season may be due to the small sample sizes.

**FIGURE 6:**
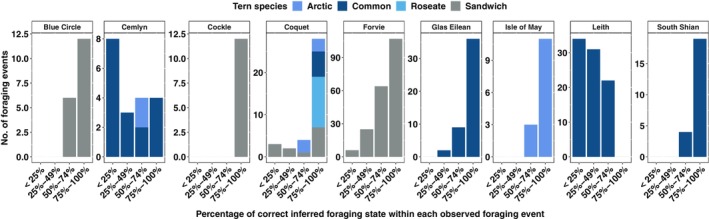
Proportion of correctly inferred foraging states within each observed foraging event across the study sites during chick‐rearing.

**FIGURE 7:**
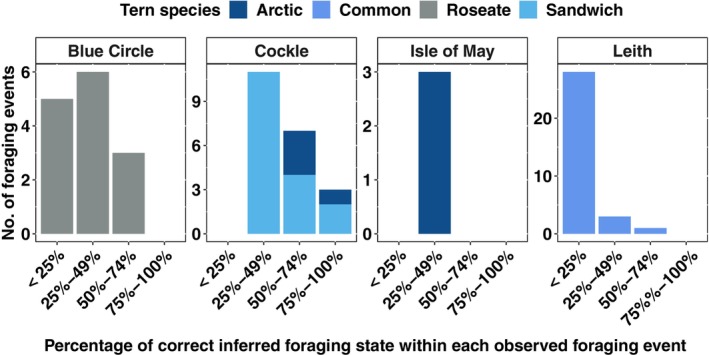
Proportion of correctly inferred foraging states within each observed foraging event across study sites during incubation.

**FIGURE 8:**
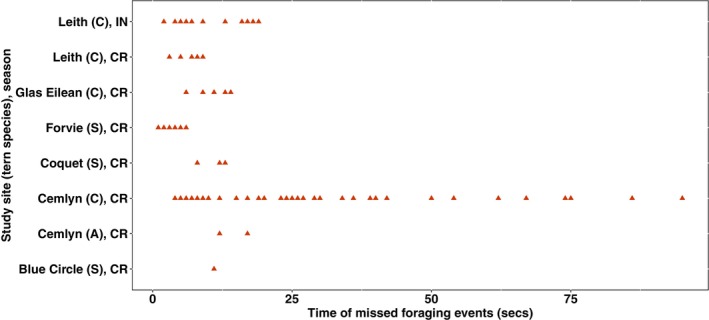
Observed foraging events completely missed from inferred foraging events across the study sites during chick‐rearing (CR) and incubation (IN). A, Arctic; C, Common; S, Sandwich tern.

Examining the corresponding observed behavioural data for each movement track of the boat, we identified and defined a foraging bout within each track where observed foraging behaviours were recorded as a foraging event. Optimal models correctly identify at least 50% of foraging behaviour within each observed foraging event, most times during chick‐rearing (Figure 6). The reverse is, however, the case during incubation (Figure 7). The number of observed foraging events completely missed across study sites (i.e. observed foraging events where the model infers foraging at 0% of the time points) sums to 65, with a median time of 13 s (Figure 8).

**FIGURE 9:**
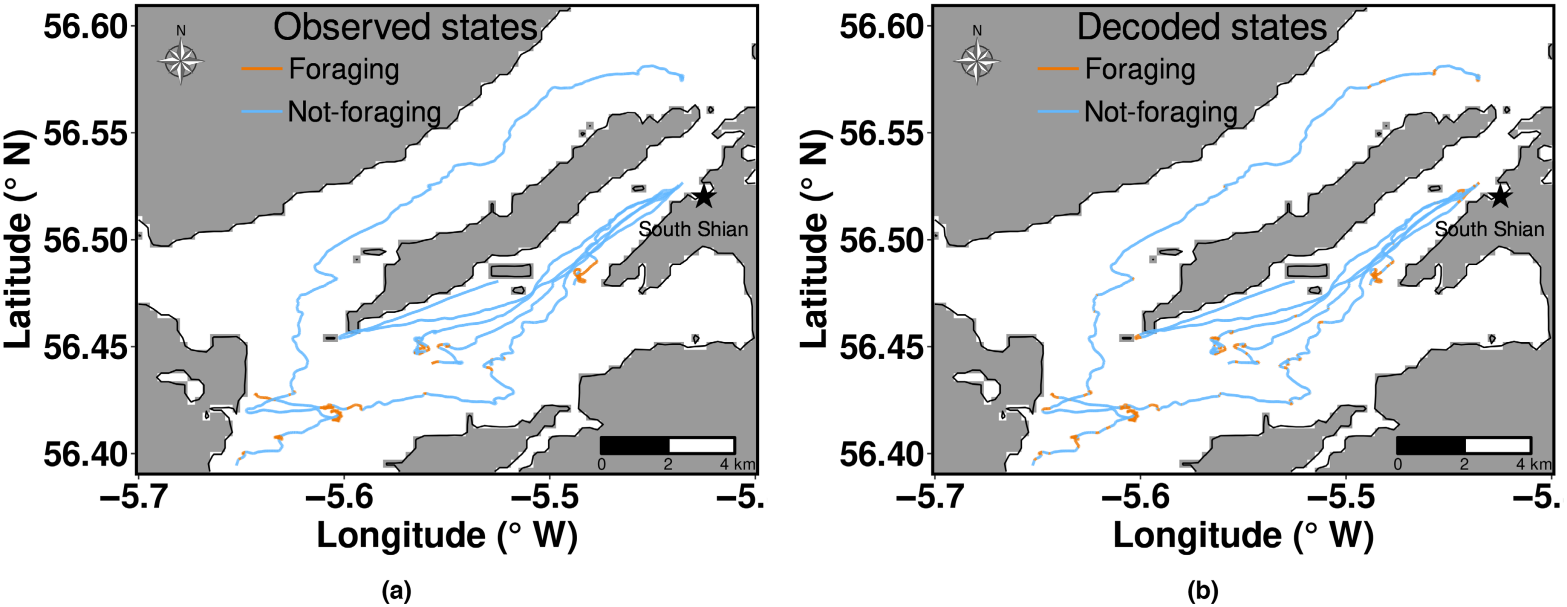
Visual tracks of six common terns coloured with (a) observed and (b) decoded behavioural states from South Shian colony during the chick‐rearing period, 2011.

**FIGURE 10:**
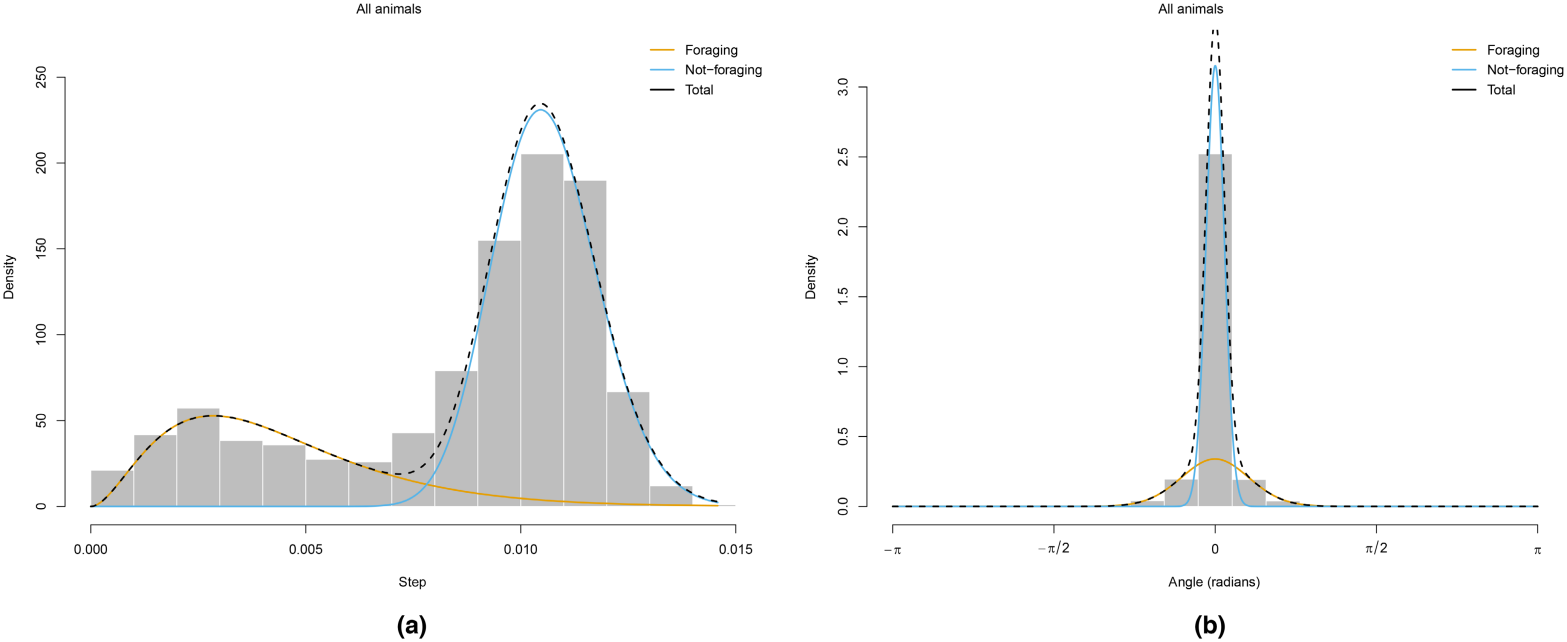
Histograms showing the distribution of (a) step length (km) and (b) turning angle of six visually (boat) tracked common terns from South Shian colony during chick‐rearing period, 2011. Lines represent HMM‐fitted state‐dependent distributions coloured according to the decoded behavioural states.

The visual tracks coloured with behavioural states (see, e.g. Figure 9) reveal similarity in the inferred and observed behavioural states across time points within visual tracking trips conducted across breeding colonies. Figure 10 provides histograms of the step length and turning angle overlaid with the density curves of the inferred behavioural states for a given track (see Figures S5–S7 for additional tracks). The inferred states assigned to foraging show shorter step lengths and lower directional persistence in turnings than the not‐foraging states, which exhibit larger step lengths and high directional persistence in turnings.

All models fitted appeared to have similar inferred states so that the inferred states were largely insensitive to the set of models considered. However, AIC identified the same, relatively complex model (e.g. an HMM with a relatively large number of model parameters) across many species and breeding colonies, while the validation metrics identified much simpler models. During incubation, we observe that the HMM accounting for the Euclidean distance of the boat to the colony as a covariate effect is mostly considered optimal compared to the chick‐rearing period. Furthermore, since there are no young terns to look after at the colony during incubation, terns are likely to forage further from the colony during this period compared to chick‐rearing. Thus, accounting for the distance of the terns to the colony in HMMs may provide better behavioural inference.

## 4 DISCUSSION

### 4.1 Visual tracking as a tool for validating HMMs

Inferred behavioural states from telemetry data have not been validated in many previous studies due to the difficulty in obtaining concurrent observed behavioural data. However, these inferred behaviours are used in ecology to delineate important areas, such as those used for foraging, and effective conservation planning and management decisions are taken based on the location of these behaviours. Given the current climate and appetite for increasing the number of protected areas on land and sea globally (e.g. protecting 30% of the earth by 2030 target from the UN Biodiversity COP 15), it is crucial to assess the validity of behaviours inferred from HMMs used in identifying the size and location of essential areas to be protected.

In practice, behavioural states of seabirds are mostly inferred from HMMs fitted to telemetry data (Langrock et al., 2012), and our study is the first to infer behavioural states of seabirds from visual tracking data using HMMs. We acknowledge that there may be potential effects of the boat following the seabirds on their behaviour, the inferred states and the validation process itself. However, previous studies have shown that the visual tracking method does not unduly affect bird behaviour due to a reasonable distance maintained between the individuals and the boat; moreover, most birds appear to ignore the boat (Perrow et al., 2011; Robertson et al., 2014; Wilson et al., 2014). The distance between the boat and the bird was, however, increased when there was a noticeable change in behaviour, such as evasive flight, observed for a few birds (Perrow et al., 2011, Robertson et al., 2014, Wilson et al., 2014). These previous studies did not investigate the extent to which boat‐based tracks replicate the path taken by the birds. Our study shows that movement data from the boat being used to visually track terns closely replicated those from the estimated location data of the terns being tracked, particularly for movement tracks corresponding to the foraging behavioural states of terns. Additionally, similar behavioural states of terns were inferred from HMMs fitted to the boat tracks and the corresponding actual (estimated) tern location data. We acknowledge that the boat and approximate tern position were compared for a small number of tern species and restricted to a single colony (Coquet) and breeding period (chick‐rearing), which may impose limitations on the how representative the data and how generalised the interpretation of the results can be. However, there are previous studies where individual terns were tracked visually using a boat with tracks obtained from the onboard GPS as proxies for foraging tracks have been used to successfully identify foraging behaviours and areas of tern species (Perrow et al., 2011, Wilson et al., 2014).

The unique approach of the visual tracking method provides telemetry data for the boat, a proxy for the tracks of the terns they are following and additional behavioural observation data, which are difficult to access in terrains such as the marine environment. Consequently, it allows inferred behaviours of seabirds to be validated using behavioural observations.

### 4.2 Validating inferred behavioural states

Our study investigated the accuracy of HMMs fitted to visual tracking data from different tern species across breeding colonies in the United Kingdom during the breeding season, using behavioural observation data recorded by observers on the boats. Results suggest that HMMs can correctly infer behavioural states from tracking data. A similar observation has been shown for inferred behavioural states from HMMs using additional accelerometer and magnetometer data from four species of albatross Conners et al. (2021) and fishermen's movement data with frequency differing from the observed behaviours (Joo et al., 2013). These methods used to infer behaviours are subject to the accuracy of the measurement devices. Our study is the first to validate HMMs using observed behaviours taken concurrently as the tracking data in the same spatial and temporal context. Generally, HMMs performed reasonably well at decoding behavioural states. However, the performance during incubation was poor compared to chick‐rearing, particularly for Arctic terns at the Isle of May (42% see Table 3). Terns on the Isle of May had reduced breeding success in 2010. Therefore, terns that were tracked may have included failed or non‐breeders which are not required to return to the colony regularly to attend to eggs or chicks, and so the data for this colony and year may be potentially unrepresentative of breeding adults (Wilson et al., 2014).

The capacity of HMMs in identifying and capturing most foraging behavioural activities within a foraging bout was low for roseate terns at Blue Circle and common terns at Leith during incubation in 2010 (Figure 6) and common terns at Cemlyn and Leith during chick‐rearing (Figure 7). The visual tracking method was aimed at chick‐rearing (2009–2011) but was extended to incubation in 2010, resulting in a reduction in the frequency of data collection (through survey effort being split between time periods) (Wilson et al., 2014), which may be a potential reason for the poor performance of fitted HMMs during incubation and chick‐rearing in 2010. Observed behavioural data showed that common terns at Leith colony foraged closer to the colony during chick‐rearing, 2010 (Figure 2b). The Leith common tern colony is in a port, so there may have been speed restrictions on the boat and limitations to how well the boat could closely replicate the movement of the terns. It is unclear from our study the exact reason why fitted HMMs did not identify most foraging behavioural states of common terns within foraging events at Leith and Cemlyn. However, overall, 70% (Leith) and 81% (Cemlyn) of the foraging behavioural states were decoded correctly from HMMs.

HMMs inferred foraging behavioural states 0% of the time for some observed foraging events that lasted for an average of 21 s. These missed foraging events were most common in chick‐rearing. Terns forage close to the colony during chick‐rearing and do not travel for long distances (as they do in incubation) (Eglington & Perrow, 2014). Also, observers noted short sessions of foraging behavioural activities of some tracked terns in some colonies (JNCC personal communication). As a result, the track of the boat may not capture tern movement corresponding to these short observed foraging events. Consequently, boat tracks may not have represented the tern's track correctly within those short phases of foraging events. As such, the HMMs fitted to boat tracks from such a scenario could not have decoded foraging states within the foraging bout from the boat tracks.

The choice of the number of behavioural states to fit in HMMs is a major challenge in animal movement modelling particularly when the goal is to infer behavioural states from telemetry data. AIC tends to select HMMs with more states but may not correspond to or have a meaningful biological interpretation of the studied animal. Pohle et al. (2017) provides practical guides in selecting the number of states to fit HMMs. Given a fixed number of states, an additional model selection process may include covariates or consider pooling across individual tracks. However, within our study, these different models did not lead to any substantial differences between the inferred behavioural states, as identified by McClintock (2021). Therefore, fitting less complex HMMs may likely outperform complex models in inferring hidden behavioural states from movement data. As such, when behavioural inference is the study's goal, it may be preferable to consider simpler models (i.e. including a smaller number of model parameters) when choosing an appropriate HMM to fit after selecting the desired number of states.

Our findings have relevance to conservation management and planning. Seabird colonies are more likely to be included as part of protected area networks due to their aggregated nature and relative ease of delineation than areas used by seabirds at sea, especially for species with large foraging ranges from the colony. Foraging areas are considered important habitats to include within seabird‐protected area networks (Lascelles et al., 2016). Thus, foraging behavioural activities can be a focus for future studies looking at using behavioural states to inform conservation and management, such as identifying the optimal size and location of foraging areas around seabird colonies. In addition, our study could be extended to assess how temporal validation translates to spatial validation. The visual tracking data could be used to compare the spatial distribution of behaviours inferred from HMMs with the spatial distribution of observed behaviours to determine the accuracy of foraging areas detected using HMMs with real‐world implications for conservation and management.

Our study provides evidence, in the context of UK tern populations, that using HMMs to infer foraging behavioural states can help identify most foraging events correctly, and it would be valuable to explore in future work whether this result can be generalised to other taxa and geographical areas. Additionally, we provide evidence that when a 2‐state HMM is implemented (interpreted for the UK terns as foraging and not foraging) most foraging and non‐foraging events are identified correctly by the chosen model. Future work can be explored to determine whether this would hold true for three or more states for UK terns, and whether the result could be generalised to other species and taxa in three or more state HMMs. Missed foraging events or bouts may be less frequent from HMMs fitted to telemetry data of seabirds as GPS devices attached to seabirds are more likely to capture movement patterns influenced by short foraging behavioural activities that last a short time than HMMs fitted to visual tracking data. Therefore, using HMMs for behavioural inference, particularly the foraging behaviour of seabirds, can aid spatial planning and inform conservation decisions, hence providing a tool for the effective management of the impact of human activities on seabirds and other species.

In summary, using HMMs to infer important conservation‐relevant behaviours from telemetry data appears defensible for UK tern populations based on our results, suggesting that they can be used to inform the design of designated protected areas. A priority for future research would be to explore the extent to which these results are generalisable. Understanding how generalisable the results from this study are to other taxa or species would require appropriate validation data which are collected contemporaneously with tracking information on individuals. We therefore recommend that observational behaviour data that can be used as a ‘gold standard’ for validation should be collected, wherever feasible, alongside GPS data. Lastly, there is evidence from our validation study that given the same number of behavioural states, there may be no substantial differences in the performance of simpler and complex HMMs in inferring behavioural states even in situations where standard model selection approaches, such as AIC, strongly suggest the use of more complex models.

## AUTHOR CONTRIBUTIONS

**Rebecca A. Akeresola:** Data curation (equal); formal analysis (lead); funding acquisition (equal); methodology (equal); software (lead); validation (lead); visualization (lead); writing – original draft (lead); writing – review and editing (equal). **Adam Butler:** Conceptualization (equal); funding acquisition (equal); methodology (supporting); supervision (equal); validation (supporting); writing – original draft (supporting); writing – review and editing (supporting). **Esther L. Jones:** Conceptualization (equal); funding acquisition (equal); methodology (supporting); supervision (equal); validation (supporting); writing – original draft (supporting); writing – review and editing (supporting). **Ruth King:** Conceptualization (equal); funding acquisition (equal); methodology (supporting); supervision (equal); validation (supporting); writing – original draft (supporting); writing – review and editing (supporting). **Víctor Elvira:** Conceptualization (equal); funding acquisition (equal); methodology (supporting); supervision (equal); validation (supporting); writing – original draft (supporting); writing – review and editing (supporting). **Julie Black:** Data curation (lead); writing – original draft (supporting); writing – review and editing (supporting). **Gail Robertson:** Conceptualization (equal); funding acquisition (equal); methodology (supporting); supervision (equal); validation (supporting); writing – original draft (supporting); writing – review and editing (supporting).

## CONFLICT OF INTEREST STATEMENT

All authors have no conflict of interest to declare.

## Supporting information


Appendix S1

## Data Availability

Visual tracking data used in this work is provided by the Joint Nature Conservation Committee (JNCC). The data and code are contained in the repository: https://github.com/aotara/Validating‐HMMs‐project. The visual tracks for tern species in Coquet Island Colony are already published in https://hub.jncc.gov.uk/assets/0de5aa81‐6aa1‐4d33‐a239‐4484c5b13573# while the visual tracks for other colonies considered in our article will be published.
