# Supplementary material for: Validating hidden Markov models for seabird behavioural inference
Source: Ecol Evol. 2024 Mar 4;14(3):e11116. doi: 10.1002/ece3.11116 (PMC10911961; doi:10.1002/ece3.11116)
Supplement: Supplementary file 1 — Appendix S1 [file ECE3-14-e11116-s001.pdf]

Supporting Information
Validating hidden Markov models for seabird behavioural inference

Rebecca A. Akeresola[1,2], Adam Butler[2], Esther L. Jones[2], Ruth King[1], Víctor Elvira[1], Julie Black[3], and Gail Robertson[2]

[1]School of Mathematics and Maxwell Institute for Mathematical Sciences, University of Edinburgh, Edinburgh, UK
[2]Biomathematics & Statistics Scotland, Edinburgh, UK
[3]Joint Nature Conservation Committee, UK

Table 1: Summary of visual tracking data of terns across the study sites.

Colony, Year	Tern species	Number of tracks	Number of locations
Incubation period			
Blue Circle, 2010	roseate	1	9236
Cockle, 2010	Arctic	1	1793
	Sandwich	3	16076
Isle of May, 2010	Arctic	2	8997
Leith, 2010	common	6	15077
Chick-rearing period			
Cemlyn, 2009	Arctic	2	2861
	common	15	23328
Blue Circle, 2010	Sandwich	9	13167
Cockle, 2010	Sandwich	5	13014
Coquet, 2010	Arctic	5	10793
	common	2	2384
	roseate	1	2984
	Sandwich	3	6684
Isle of May, 2010	Arctic	5	3292
Leith, 2010	common	17	34681
Eilean Glas, 2011	common	15	25991
Forvie, 2011	Sandwich	15	38747
South Shian, 2011	common	6	14272

Assessment of visual tracking data as a proxy for tern tracking data

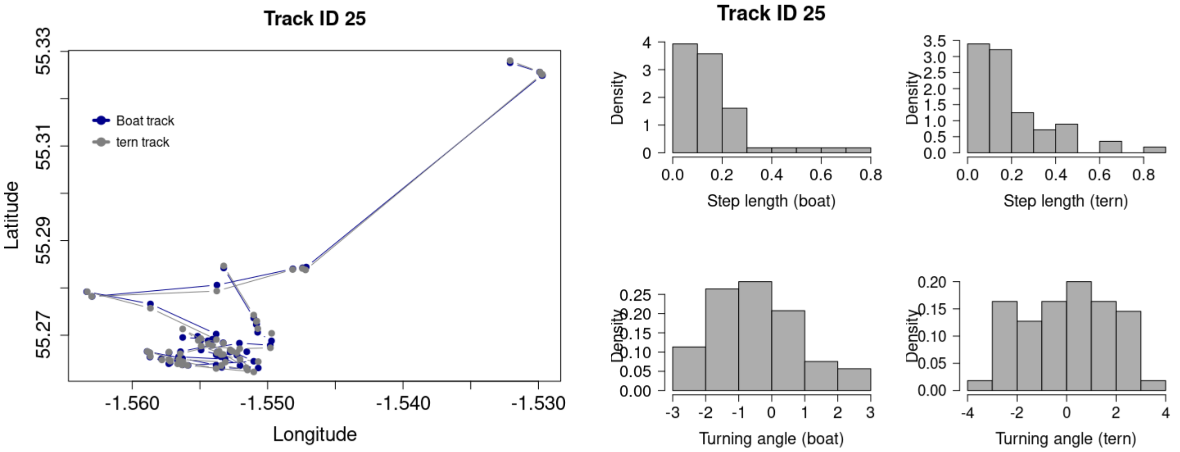

(a) (b)

Figure S1: (a) Approximate Arctic tern tracks and corresponding boat tracks. (b) Histogram showing the distribution of step length (km) from boat tracks (top left) and approximate Arctic tern tracks (top right); and histogram showing the distribution of turning angle (radians) from boat tracks (bottom left) and approximate Arctic tern tracks (bottom right) from Coquet Island, 2009.

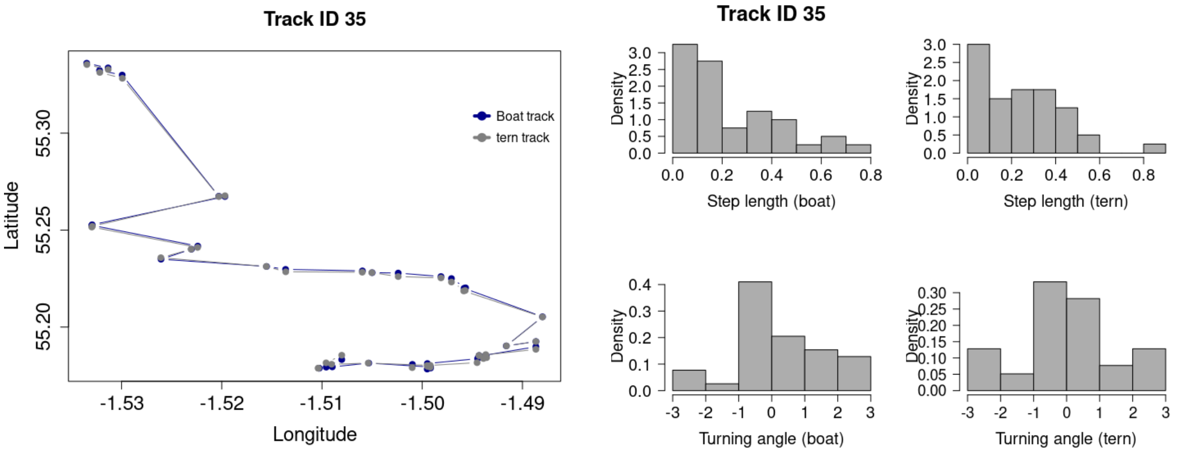

(a) (b)

Figure S2: (a) Approximate Sandwich tern tracks and corresponding boat tracks. (b) Histogram showing the distribution of step length (km) from boat tracks (top left) and approximate Sandwich tern tracks (top right); and histogram showing the distribution of turning angle (radians) from boat tracks (bottom left) and approximate Sandwich tern tracks (bottom right) from Coquet Island, 2009.

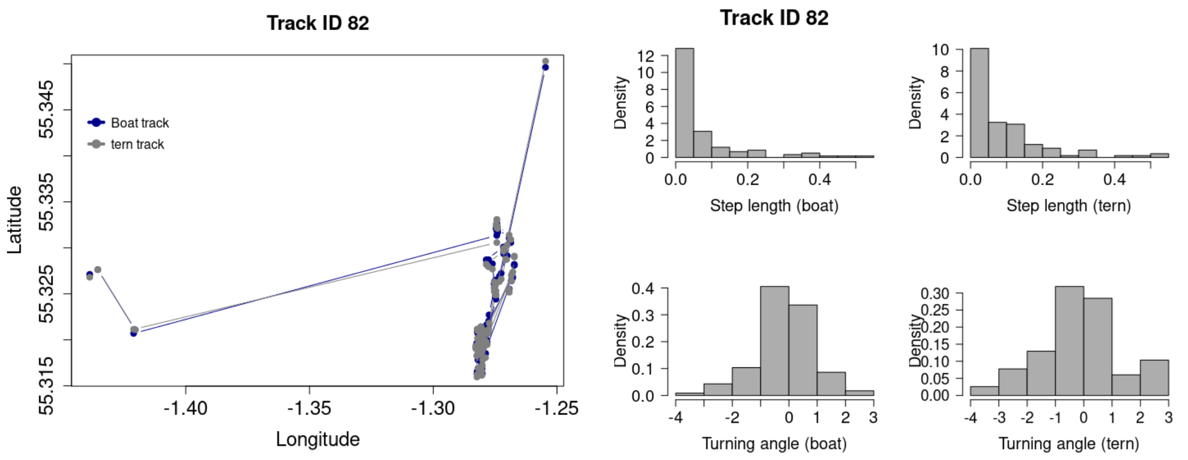

(a) (b)

Figure S3: (a) Approximate Arctic tern tracks and corresponding boat tracks. (b) Histogram showing the distribution of step length (km) from boat tracks (top left) and approximate Arctic tern tracks (top right); and histogram showing the distribution of turning angle (radians) from boat tracks (bottom left) and approximate Arctic tern tracks (bottom right) from Coquet Island, 2009.

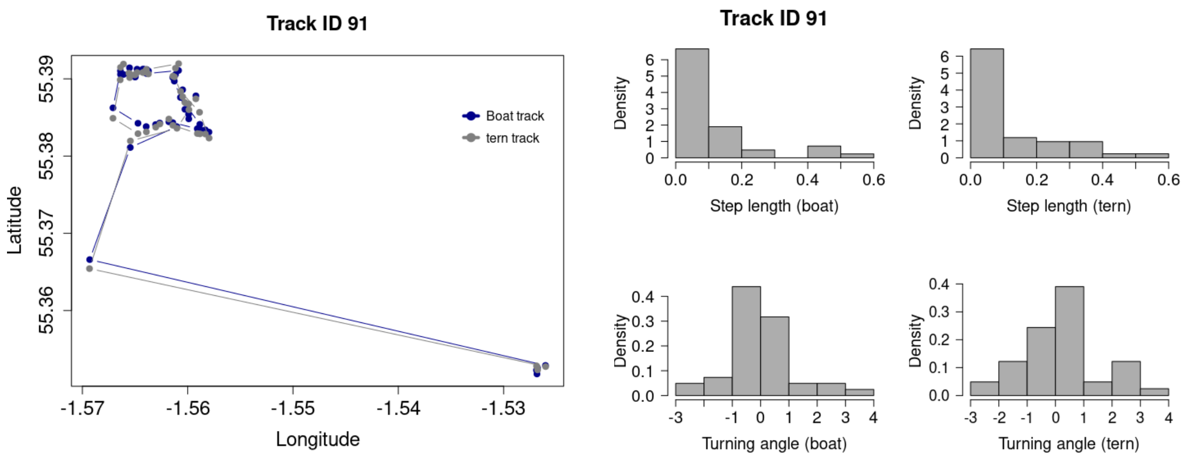

(a) (b)

Figure S4: (a) Approximate common tern tracks and corresponding boat tracks. (b) Histogram showing the distribution of step length (km) from boat tracks (top left) and approximate common tern tracks (top right); and histogram showing the distribution of turning angle (radians) from boat tracks (bottom left) and approximate common tern tracks (bottom right) from Coquet Island, 2009.

Validating HMM-inferred behavioural states

Table 2: Validation results of HMMs fitted to terns visual tracks from Cemlyn, Cockle, and Coquet colonies during the chick-rearing period. nPar = number of model parameter, PPV = positive predictive value (foraging), NPV = negative predictive value (not-foraging), TPR = true positive rate. HMM deemed optimal based on *AIC and ** validation (lowest log-loss value).

Chick-rearing, 2010					Validation metrics				
Colony	Species	Model	nPar	ΔAIC	PPV	NPV	TPR	F1-score	log-loss
Cemlyn	Arctic	0	13	1066	0.9891	0.2676	0.5884	0.7378	12.4048
		1	15	1062	0.9904	0.2677	0.5863	0.7365	12.4077
		2*	19	**0**	0.9740	0.2562	0.5904	0.7351	12.7527
		3	21	1	0.9740	0.2562	0.5904	0.7351	12.7356
		4**	15	1120	0.9891	0.2680	0.5892	0.7385	**12.3897**
		5	19	1599	0.9891	0.2522	0.5528	0.7092	13.5970
		6	21	991	0.9891	0.2659	0.5847	0.7349	12.7588
	Common	0	13	7308	0.7179	0.8667	0.7974	0.7555	6.7611
		1	41	7191	0.7189	0.8664	0.7966	0.7557	6.7450
		2	97	58	0.6806	0.8623	0.7996	0.7353	7.6093
		3*	125	**0**	0.6825	0.8638	0.8016	0.7372	7.5658
		4	15	8824	0.7190	0.8668	0.7972	0.7560	6.7481
		5	19	13169	0.7187	0.8589	0.7824	0.7491	6.9283
		6**	21	8688	0.7193	0.8749	0.8121	0.7629	**6.6618**
Cockle	Sandwich	0	13	6739	0.8776	0.9531	0.8602	0.8688	2.3079
		1	21	6709	0.8770	0.9531	0.8602	0.8685	2.3154
		2*	37	**0**	0.8428	0.9691	0.9111	0.8756	2.2514
		3**	45	136	0.8485	0.9692	0.9111	0.8787	**2.1887**
		4	15	6737	0.8776	0.9531	0.8602	0.8688	2.3076
		5	19	5819	0.8810	0.9487	0.8459	0.8630	2.3499
		6	21	5818	0.8810	0.9487	0.8459	0.8630	2.3486
Coquet	Arctic	0	13	3286	0.6657	0.8309	0.6219	0.6430	7.5728
		1**	21	3089	0.6684	0.8298	0.6175	0.6419	**7.5243**
		2	37	233	0.5965	0.7840	0.4941	0.5404	9.1235
		3*	45	**0**	0.6359	0.7904	0.5003	0.5600	8.6709
		4	15	3449	0.6636	0.8295	0.6184	0.6402	7.6335
		5	19	4693	0.6607	0.8298	0.6202	0.6398	7.7781
		6	21	4676	0.6607	0.8298	0.6202	0.6398	7.7790
	Common	0**	13	140	0.8844	0.8738	0.7919	0.8356	**4.1822**
		1	15	110	0.8851	0.8766	0.7972	0.8388	4.2520
		2	19	37	0.8803	0.8779	0.8004	0.8384	4.2706
		3*	21	**0**	0.8750	0.8770	0.7994	0.8354	4.3348
		4	15	244	0.8870	0.8762	0.7962	0.8391	4.2204
		5	19	596	0.8886	0.8741	0.7919	0.8374	4.2700
		6	21	595	0.8886	0.8741	0.7919	0.8374	4.2677
	Roseate	0*	11	**0**	0.6807	0.9030	0.8372	0.7508	6.6749
		4**	13	99	0.6866	0.9157	0.8606	0.7638	**6.6383**
		5	15	547	0.7031	0.8908	0.8080	0.7519	6.9007
		6	17	550	0.7048	0.8896	0.8051	0.7516	6.9086
	Sandwich	0	13	2779	0.9087	0.6885	0.7010	0.7914	7.4168
		1	17	2755	0.9074	0.6880	0.7008	0.7908	7.4247
		2	25	27	0.8703	0.7105	0.7474	0.8041	7.2977
		3**,*	25	**0**	0.8690	0.7103	0.7477	0.8038	**7.2869**
		4	15	2882	40.9069	0.6882	0.7013	0.7909	7.4101
		5	19	3950	0.9057	0.6713	0.6764	0.7744	8.0292
		6	21	2828	0.9088	0.6840	0.6943	0.7871	7.5363

Table 3: Validation results of HMMs fitted to terns visual tracks from Blue Circle, Forvie, Glas Eileanan, Isle of May, Leith, and South Shian colonies during the chick-rearing breeding season. nPar = number of parameter, PPV = positive predictive value (foraging), NPV = negative predictive value (not-foraging), TPR = true positive rate. HMM deemed optimal based on *AIC and ** validation (lowest-log-loss value).

Chick-rearing					Validation metrics				
Colony	Species	Model	nPar	ΔAIC	PPV	NPV	TPR	F1-score	log-loss
2010									
Blue Circle	Sandwich	0	13	4139	0.7930	0.8105	0.7780	0.7854	6.8522
		1	29	4102	0.7928	0.8106	0.7782	0.7854	6.8540
		2**	61	47	0.8084	0.8171	0.7836	0.7958	**6.4905**
		3*	77	**0**	0.8075	0.8170	0.7837	0.7954	6.4979
		4	15	4090	0.7920	0.8110	0.7791	0.7854	6.8483
		5	19	3860	0.7867	0.8058	0.7728	0.7796	7.0015
		6	21	3818	0.7893	0.8057	0.7718	0.7804	6.9874
Isle of May	Arctic	0	13	776	0.8641	0.7170	0.7099	0.7794	7.5165
		1**	21	761	0.8677	0.7158	0.7065	0.7788	**7.5086**
		2*	37	**0**	0.8440	0.7021	0.6953	0.7624	8.0858
		3	45	5	0.8440	0.7021	0.6953	0.7624	8.0846
		4	15	779	0.8641	0.7170	0.7099	0.7794	7.5142
		5	19	750	0.8652	0.7148	0.7059	0.7774	7.5414
		6	21	753	0.8652	0.7148	0.7059	0.7774	7.5409
Leith	Common	0	13	9960	0.7439	0.8014	0.6541	0.6961	7.5829
		1	45	9812	0.7435	0.7995	0.6498	0.6934	7.6054
		2**	109	50	0.7405	0.8218	0.7019	0.7207	**7.2188**
		3*	141	**0**	0.7397	0.8194	0.6969	0.7176	7.2345
		4	15	9904	0.7439	0.8012	0.6537	0.6958	7.5852
		5	19	8039	0.7436	0.7936	0.6356	0.6853	7.7034
		6	21	7988	0.7441	0.7937	0.6356	0.6855	7.7042
2011									
Forvie	Sandwich	0	13	8483	0.6398	0.8912	0.8047	0.7128	7.3545
		1	41	8205	0.6406	0.8906	0.8033	0.7127	7.3447
		2	97	146	0.6509	0.8904	0.8001	0.7178	7.1520
		3**,*	125	**0**	0.6515	0.8899	0.7990	0.7177	**7.1351**
		4	15	10766	0.6413	0.8906	0.8030	0.7130	7.3408
		5	19	15588	0.6450	0.8880	0.7963	0.7127	7.3844
		6	21	10760	0.6460	0.8884	0.7970	0.7135	7.2615
Glas Eileanan	Common	0**	13	8929	0.8421	0.8174	0.7349	0.7848	**6.0409**
		1	41	8338	0.8451	0.8140	0.7278	0.7820	6.0940
		2	97	182	0.7989	0.8296	0.7676	0.7829	6.4072
		3*	125	**0**	0.8136	0.8272	0.7595	0.7856	6.2590
		4	15	9662	0.8425	0.8186	0.7369	0.7861	6.0485
		5	19	14241	0.8378	0.8069	0.7161	0.7721	6.3626
		6	21	9579	0.8467	0.8135	0.7264	0.7819	6.0473
South Shian	Common	0	13	4459	0.7026	0.9668	0.8995	0.7889	3.9145
		1	23	4432	0.6996	0.9667	0.8995	0.7870	3.9328
		2	43	2	0.6823	0.9630	0.8890	0.7720	4.2433
		3*	53	**0**	0.6842	0.9631	0.8890	0.7732	4.2309
		4**	15	4796	0.7051	0.9690	0.9064	0.7932	**3.8743**
		5	19	8286	0.7023	0.9620	0.8841	0.7827	4.0401
		6	21	8286	0.7021	0.9612	0.8817	0.7817	4.0353

Table 4: Validation results of HMMs fitted to terns visual tracks from Blue circle, Cockle, Isle of May, and Leith colonies during incubation breeding season. nPar = number of model parameter, PPV = positive predictive value (foraging), NPV = negative predictive value (not-foraging), TPR = true positive rate. HMM deemed optimal based on *AIC and ** validation (lowest log-loss value).

Incubation, 2010		Validation metrics							
Colony	Species	Model	nPar	ΔAIC	PPV	NPV	TPR	F1-score	log-loss
Blue Circle	Roseate	0	13	811	0.8291	0.4181	0.6057	0.7000	12.8071
		4**	15	782	0.8290	0.4182	0.6062	0.7003	**12.7875**
		5	19	14	0.8217	0.4059	0.5900	0.6868	13.1571
		6*	21	**0**	0.8218	0.4057	0.5893	0.6863	13.1520
Cockle	Arctic	0	13	332	0.6071	0.7925	0.6667	0.6355	10.0929
		4	15	329	0.6109	0.7977	0.6772	0.6423	10.0102
		5**	19	11	0.6011	0.7992	0.6863	0.6408	**9.7765**
		6*	21	**0**	0.6000	0.7996	0.6878	0.6409	9.7904
	Sandwich	0	13	2293	0.5955	0.7074	0.4915	0.5385	11.3537
		1	17	2093	0.5957	0.7073	0.4909	0.5382	11.3726
		2	25	224	0.5910	0.7026	0.4785	0.5288	11.4727
		3*	29	**0**	0.5899	0.7007	0.4727	0.5248	11.4857
		4	15	2294	0.5955	0.7074	0.4915	0.5385	11.3536
		5	19	1437	0.5957	0.7087	0.4959	0.5412	11.3433
		6**	21	1433	0.5954	0.7088	0.4965	0.5415	**11.3388**
Isle of May	Arctic	0	13	611	0.6341	0.6548	0.3283	0.4326	12.1206
		1	15	598	0.6351	0.6549	0.3283	0.4328	12.1236
		2	19	312	0.6278	0.6535	0.3269	0.4299	12.1224
		3	21	299	0.6284	0.6539	0.3283	0.4312	12.1207
		4	15	544	0.6315	0.6543	0.3280	0.4317	12.1213
		5	19	82	0.6375	0.6527	0.3174	0.4237	12.0617
		6**,*	21	**0**	0.6388	0.6539	0.3220	0.4282	**12.0572**
Leith	Common	0**	13	1071	0.6100	0.8352	0.6029	0.6064	**7.9425**
		1	23	1045	0.6099	0.8352	0.6028	0.6063	7.9465
		2	43	22	0.6082	0.8353	0.6039	0.6060	8.0407
		3*	53	**0**	0.6076	0.8353	0.6041	0.6058	8.0435
		4	15	994	0.6110	0.8355	0.6035	0.6072	7.9548
		5	19	555	0.6113	0.8381	0.6120	0.6116	7.9488
		6	21	476	0.6102	0.8373	0.6098	0.6099	7.9644

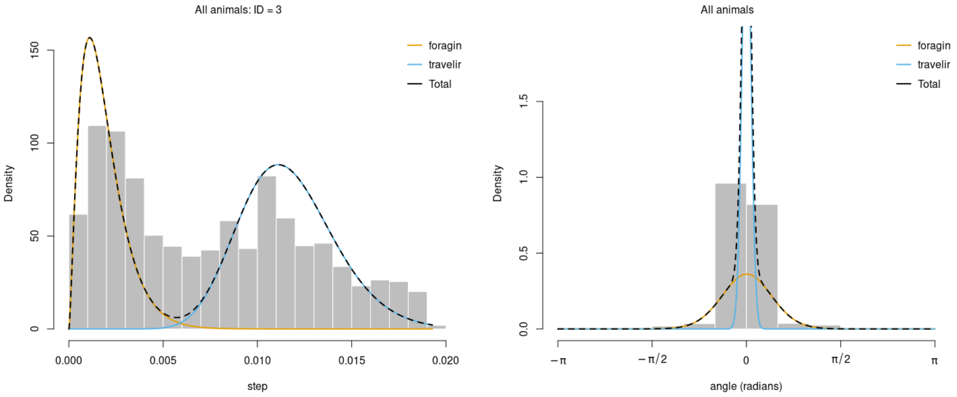

(a) (b)

Figure S5: Histograms showing the distribution of (a) step length (km) and (b) turning angle of 9 visually tracked Sandwich terns from Blue Circle colony during chick-rearing period, 2010. Lines represent HMM-fitted state-dependent distributions and are coloured according to the decoded behavioural states foraging (orange line) and not-foraging (blue line).

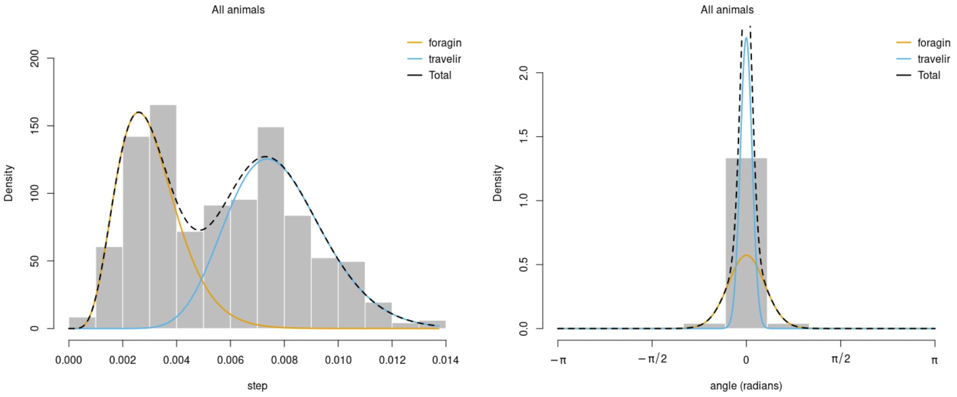

(a) (b)

Figure S6: Histograms showing the distribution of (a) step length (km) and (b) turning angle of 5 visually tracked Arctic terns from Isle of May colony during chick-rearing period, 2010. Lines represent HMM-fitted state-dependent distributions and are coloured according to the decoded behavioural states foraging (orange line) and not-foraging (blue line).

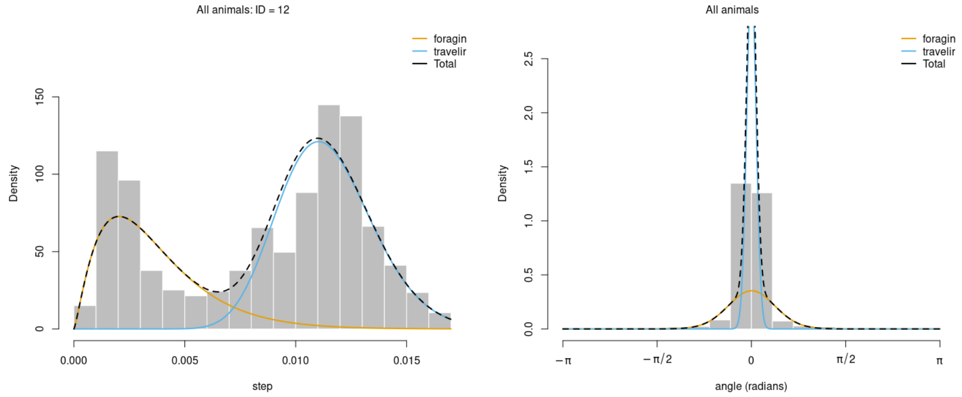

(a) (b)

Figure S7: Histograms showing the distribution of (a) step length (km) and (b) turning angle of 2 visually tracked common terns from Coquet Island colony during chick-rearing period, 2010. Lines represent HMM-fitted state-dependent distributions and are coloured according to the decoded behavioural states foraging (orange line) and not-foraging (blue line).
